# Genetic Divergence in Northern Benin Sorghum (*Sorghum bicolor* L. Moench) Landraces as Revealed by Agromorphological Traits and Selection of Candidate Genotypes

**DOI:** 10.1155/2015/916476

**Published:** 2015-02-01

**Authors:** Innocent Dossou-Aminon, Laura Yêyinou Loko, Arlette Adjatin, Eben-Ezer B. K. Ewédjè, Alexandre Dansi, Sujay Rakshit, Ndiaga Cissé, Jagannath Vishnu Patil, Clément Agbangla, Ambaliou Sanni, Akpovi Akoègninou, Koffi Akpagana

**Affiliations:** ^1^Laboratory of Biotechnology, Genetic Resources and Plant and Animal Breeding (BIORAVE), Faculty of Sciences and Technology of Dassa, Polytechnic University of Abomey, 01 BP 14 Dassa-Zoumè, Benin; ^2^Directorate of Sorghum Research, Rajendra Nagar, Hyderabad 500030, India; ^3^Regional Study Centre for Improving Adaptation to Drought (CERAAS), BP 3320, Road of Khombole, Thiès, Senegal; ^4^Laboratory of Genetics and Biotechnology, Faculty of Sciences and Techniques (FAST), University of Abomey-Calavi (UAC), Abomey-Calavi, 01 BP 526 Cotonou, Benin; ^5^Laboratory of Biochemistry and Molecular Biology, Faculty of Sciences and Technology (FAST), University of Abomey-Calavi (UAC), 01 BP 526 Cotonou, Benin; ^6^National Herbarium, Department of Botany and Plant Biology, Faculty of Sciences and Technology (FAST), University of Abomey-Calavi (UAC), 01 BP 526 Cotonou, Benin; ^7^Laboratory of Botany, Faculty of Sciences (FS), University of Lomé, P.O. Box 1515, Lomé, Togo

## Abstract

Sorghum [*Sorghum bicolor* (L.) Moench] is an important staple food crop in northern Benin. In order to assess its diversity in Benin, 142 accessions of landraces collected from Northern Benin were grown in Central Benin and characterised using 10 qualitative and 14 quantitative agromorphological traits. High variability among both qualitative and quantitative traits was observed. Grain yield (0.72–10.57 tons/ha), panicle weight (15–215.95 g), days to 50% flowering (57–200 days), and plant height (153.27–636.5 cm) were among traits that exhibited broader variability. Correlations between quantitative traits were determined. Grain yield for instance exhibited highly positive association with panicle weight (*r* = 0.901,  *P* = 0.000) and 100 seed weight (*r* = 0.247,  *P* = 0.000). UPGMA cluster analysis classified the 142 accessions into 89 morphotypes. Based on multivariate analysis, twenty promising sorghum genotypes were selected. Among them, AT41, AT14, and AT29 showed early maturity (57 to 66 days to 50% flowering), high grain yields (4.85 to 7.85 tons/ha), and shorter plant height (153.27 to 180.37 cm). The results obtained will help enhancing sorghum production and diversity and developing new varieties that will be better adapted to the current soil and climate conditions in Benin.

## 1. Introduction

Sorghum [*Sorghum bicolor* (L.) Moench] is a suitable crop for dry land farming agriculture owing to its wide adaptability and tolerance to adverse conditions as compared to other food crops. Its relative tolerance to drought and heat makes it an ideal cereal for human and animal consumption in areas with extreme temperatures and minimum precipitation, especially in dry regions [1]. Sorghum is one of the healthiest and nutritious food crops in view of its richness in minerals, fibre content, and gluten-free properties [2]. In the dry regions of the world, sorghum is used as food (grain sorghum), feed (poultry and bird feed), fodder, and fuel (sweet sorghum). Besides, it provides raw material for the production of starch, dextrose syrup, alcohol, and other industrial products [3]. However, the income of Benin sorghum farmers is still at subsistence level as they face many uncertainties in production and marketing [4]. The production problems are further compounded by the relatively low prices of sorghum, as the government's marketing support is mainly directed towards maize, cotton, and rice.

Sorghum is originated and domesticated in Africa (about 5,000–8,000 years ago) where the largest diversity of both cultivated and wild sorghum is found [5, 6]. Cultivated sorghums represent five major races (Bicolor, Caudatum, Durra, Guinea, and Kafir) and 10 intermediate races, corresponding to the pairwise combination of major races according to the panicle and spikelet morphology [7, 8]. It is a diploid species with a chromosome number of 2*n* = 20 [9, 10].

In Benin sorghum is grown in a total area 109,734 ha of land with a total production of 114,750 tonnes/year [11]. Sorghum production in Benin is constrained by both agronomic and environmental aspects. Loss of genetic resources which is the basic material for varietal improvement is also matter of concern [12]. Collection and characterization of existing landraces germplasm is a prerequisite for identifying potential germplasm for varietal improvement programme and to avoid duplication in the germplasm collection. Morphological, biochemical, and molecular markers have been deployed in crop genetic resources characterization. Among these, morphological characterization is the first, easiest, and cheapest step in classifying germplasm, estimating diversity, and registering cultivar [13]. Many studies have examined patterns of genetic diversity among sorghum accessions from* ex situ* germplasm collections using qualitative and quantitative agromorphological descriptors [12–14]. In Benin, there are many traditional landraces being grown across the northern part of the country and they represent important resource for systematic sorghum improvement. However, till date, not much systematic effort has been made to assemble and characterize these genetic resources. This study aims to characterize sorghum landraces collected across northern Benin in order to

- assess the genetic diversity of sorghum in this area;
- analyse the relationship between the most discriminating morphological traits;
- identify promising accessions for key agronomic traits for breeding purposes.

## 2. Material and Methods

### 2.1. Plant Materiel and Field Evaluation

The material used was a set of 142 accessions of sorghum landraces collected from three Departments (Atakora, Borgou, and Alibori) of Northern Benin. The trial was conducted at the experimental farm of the Faculty of Sciences and Technology of Dassa during the sorghum cropping season of 2013. Dassa is located in a relatively humid agroecological zone with two rainy seasons and mean annual rainfall varying from 1,100 to 1,400 mm/year [15]. Mean annual temperatures range from 26 to 28°C [15]. Sowing was done on 25th April 2013 in a randomized complete block design (RCBD) with three replicates and in 5 m rows with an inter- and intrarow spacing of 0.75 m and 0.50 m, respectively. Standard plant protection and agronomic (NPK 120:40:00 fertilizers applied as half basal dose of nitrogen and full dose of phosphorus at the time of sowing and half nitrogen applied after one month of sowing) measures known for sorghum were followed during the cropping season [12, 13]. Data were recorded on 14 quantitative traits following Elangovan et al. [12], Rakshit et al. [13], Djè et al. [14], and Abubakar and Bubuche [16]. These were days to 50% seed germination (50% SGD, days), days to 50% flowering (50% FD, days), stem third internode girth (STING, cm), panicle width (PaW, cm), exertion length (ExL, cm), plant height (PH, cm), width of third leaf from top (WTL, cm), length of third leaf from top (LTL, cm), panicle length (PaL, cm), peduncle length (PeL, cm), panicle weight (PaWt, g), 100-seed weight (100-SWt, g), number of internodes (NIN), and grain yield (GY, tons/ha). Ten qualitative traits were also considered following Bioversity International sorghum descriptors [17]. These were panicle compactness (PaCom), peduncle shape (PeSh), glume colour (GlCol), glume hairiness (GlHai), threshability (Thres), grain covering (GrCov), grain shape dorsal view (GrShDV), number of grains per glume (NuGrGl), grain shape profile view (GrShPV), and grain colour (GrCol). The elite genotypes identified were subjected to the mass selection on three generations to purify the varieties following Rakshit and Patil [18].

### 2.2. Data Analysis

The quantitative data were first subjected to descriptive statistics (mean, standard deviation, coefficient of variation) and analysis of variance (ANOVA) using SAS ver. 9.1 [19]. In order to identify the patterns of morphological variation, principal component analysis (PCA) was conducted on correlation matrix using Minitab 14 Software and the significant loading factors (explaining ≥30% variation) were noted following Maji and Shaibu [20]. Simple Pearson moment coefficients of correlation were computed between pairs of quantitative morphological traits. To study the morphological diversity between accessions, qualitative traits were converted into scores (Table 1), studied accessions were taken as individuals, and the qualitative parameters were used as variables. Simple matching (SM) similarity coefficients were calculated and used to produce dendrogram with the Unweighted Pair-Group Method with Arithmetic Average (UPGMA) cluster analysis [21, 22] and NTSYSpc 2.2 software [23].

## 3. Results

### 3.1. Morphological Variation

**Figure 1:**
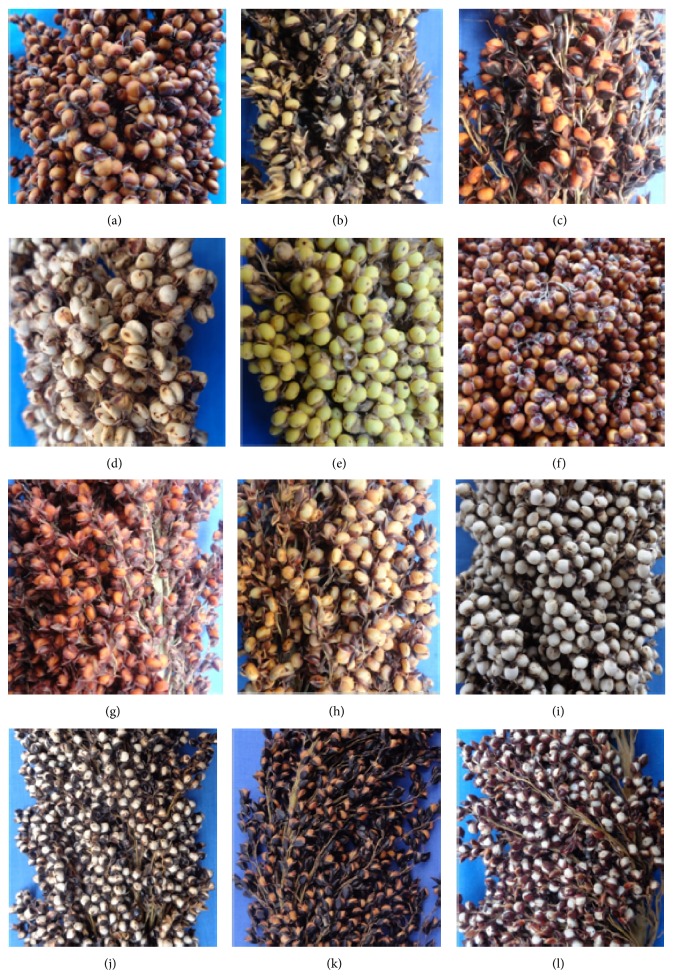
Morphological variability of grain colour, glume colour and hairiness, number of grain per glume, panicle compactness and grain covering within evaluated accessions (a) red grain, purple glume, absence of hairiness, semi-compact panicle; (b) white grain, black glume, semi-loose panicle; (c) red grain, black glume, loose panicle; (d) white grain, black glume, high hairiness, semi compact panicle, two grains per glume; (e) yellow grain, beige glume, medium hairiness, compact panicle; (f) orange grain, red glume, compact panicle; (g) red grain, red glume, 75% of grain covered, loose panicle; (h) red grain, purple glume, 50% of grain covered, semi loose panicle; (i) white grain, black glume, medium hairiness, compact panicle; (j) white grain, black glume, low hairiness, loose panicle; (k) red grain, black glume, 100% of grain covered, loose panicle; (l) white grain, purple glume, 75% of grain covered, loose panicle.

Wide variability was recorded for both qualitative and quantitative traits. Frequencies for various qualitative traits are given in Table 1. Lesser variability was recorded for number of grains per glume (97.89% of single grain), grain shape dorsal view (92.96% of dimple shape), grain covering (90.14% of 50% cover), and threshability (88.73% of easy) (Table 1). Specific morphological type was fairly well represented for peduncle shape and grain shape profile view. However, this did not prevent the significant presence of other types for these characters. The most variable traits were the panicle compactness, glume colour and hairiness, and grain colour which do not present any predominance for a particular morphological type (Figure 1).

Descriptive statistics for the 14 quantitative traits are given in Table 2. The characters like exertion length (0–44.67 cm), grain yield (0.72–10.57 tons/ha), panicle weight (15–215.95 g), and the number of internodes (7–38) were the most variable when referring to their high CV (Table 2). Among the quantitative traits, days to 50% seed germination showed the lowest variation (12.06%). The mean values of days to 50% seed germination, days to 50% flowering, panicle width, plant height, panicle length, panicle weight, 100-seed weight, and grain yield were 4.30 days, 138.56 days, 9.13 cm, 457.83 cm, 38.88 cm, 86.32 g, 3.25 g, and 3.15 tons/ha, respectively (Table 2). The minimum and maximum of days to 50% flowering, plant height, and grain yield were 57 and 200 days, 153.27 and 636.5 cm, and 0.72 and 10.57 tons/ha, respectively.

### 3.2. Principal Component Analysis (PCA) of Various Quantitative Traits

Redundancy in the data set was eliminated using the principal component analysis (PCA). Five principal components (PC1 to PC5) having eigenvalues of >1 were extracted (Table 3). These principal components accounted for most of the variability observed among the sorghum accessions collected from the different locations (73.8% of total variance). The PC1 contributed for 26.2% of the agromorphological variation followed by PC2 (19.3%), PC3 (12.1%), PC4 (9%), and PC5 (7.1%). PC1 was loaded on exertion length (37.4% of variation factor), plant height (31.7% of variation factor), width of third leaf from top (32% of variation factor), peduncle length (33.4% of variation factor), and number of internodes (36.9% of variation factor), as these traits registered above 30% variation factor [20]. With similar logic, PC2 showed a strong and positive factor with the traits like panicle weight (42.2%) and grain yield (49.5%) and a negative loading with the days to 50% flowering (30.5%). The PC3 is negatively correlated with the stem third internode girth (30.1% of variation factor), the panicle width (51.2% of variation factor), and the panicle length (56.1% of variation factor). The PC4 showed the diversity among accessions based on days to 50% seed germination (43.4% of variation factor) and 100-seed weight with negative loadings (36.3% of variation factor). PC5 confirm the agromorphological variation observed at PC4 by high positive factors (Table 3). The remaining variable had weak or no discriminatory power. Thus, the most important descriptors were those associated with PC1, PC2, PC3, and PC4.

**Figure 2:**
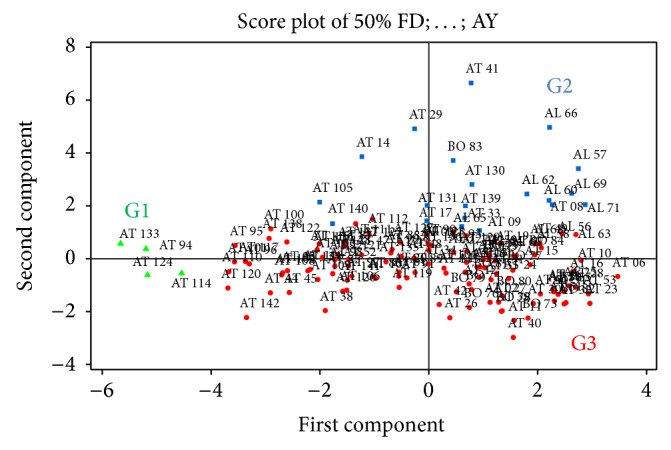
PCA grouping of the 142 Benin sorghum accessions using discriminatory quantitative traits.

Based on these descriptors, the PCA yielded three groups, namely, G1, G2, and G3 (Figure 2). G1 grouped 4 accessions taking into account exertion and peduncle length. G2 assembled 20 accessions based on grain yield, panicle weight, stem third internode girth, and 100-seed weight. G3 assembled 118 accessions based on width of third leaf from top, plant height, number of internodes, day to 50% flowering, panicle length, and panicle width.

### 3.3. Correlation between Traits and Cultivars Classification

Pearson correlation coefficients revealed significant associations between different quantitative traits evaluated (Table 4). The 50%FD had significant positive correlation with LTL (*r* = 0.120, *P* = 0.013) and highly significant positive correlation with PH (*r* = 0.606, *P* = 0.000), WTL (*r* = 0.202, *P* = 0.000), and NIN (*r* = 0.799, *P* = 0.000) but negative highly significant correlation with ExL (*r* = −0.439, *P* = 0.000) and PeL (*r* = −0.413, *P* = 0.000). Plant height showed highly significant positive correlation with STING, PaW, WTL, LTL, PaL, and NIN and significant correlation with the PaWt but highly negative correlation with PeL, ExL, and 50% SGD. The GY exhibited highly positive and significant association with STING (*r* = 0.317, *P* = 0.000), WTL (*r* = 0.125, *P* = 0.010), PaWt (*r* = 0.901, *P* = 0.000), and 100 SWt (*r* = 0.247, *P* = 0.000) but highly negative and significant correlation with ExL (*r* = −0.157, *P* = 0.001) and PeL (*r* = −160, *P* = 0.001) (Table 4).

**Figure 3:**
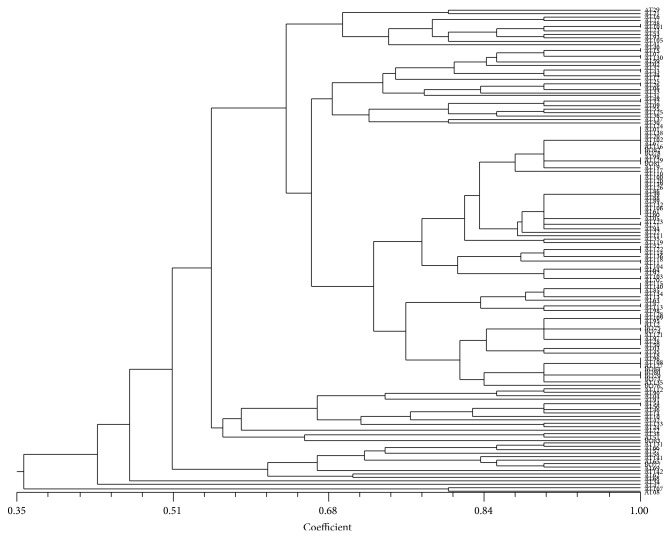
Classification of Benin sorghum accessions into 89 morphotypes using 10 qualitative traits and UPGMA clustering method.

UPGMA cluster analysis of the 142 sorghum accessions generated 89 distinct morphotypes (Figure 3). The number of accessions per morphotype varied from one to 13 accessions. Among the 89 morphotypes, 74.15% was represented by only one accession. The largest unit clusters together 13 accessions (AT110, AT100, AT120, AT139, AT126, AT86, AT39, AT88, AT89, AT132, AT106, AL61, AL60) having loose panicle, erect peduncle, black colour, low hairiness of glume, easy to thresh, 50% grain covered, dimple grains in dorsal view, single grains per glume, elliptic grain in profile view and white seeds. On the other hand four accessions (AT115, AT140, AT85, and AT134) having semiloose panicle, erect peduncle, purple colour and low hairiness of glume, easy to thresh, 50% grain covered, dimple grains in dorsal view, single grains per glume, elliptic grain in profile view, and red seed were grouped as identical.

### 3.4. Promising Genotypes for Sorghum Improvement Program in Benin

**Figure 4:**
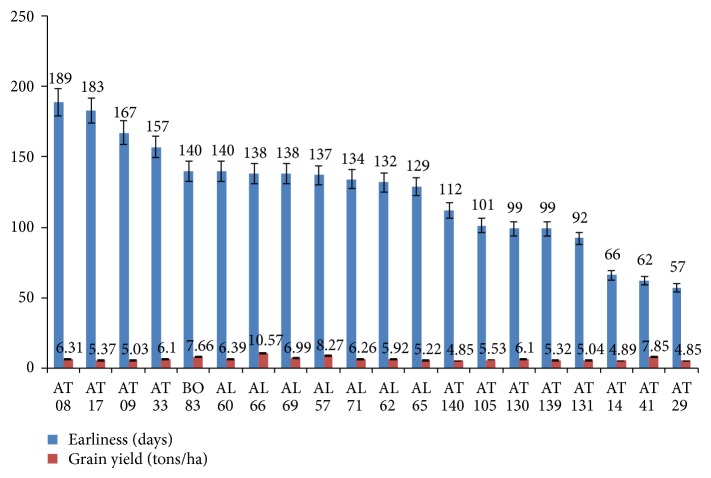
Twenty sorghum elite genotypes identified based on grain yield and days to 50% flowering.

Based on morphoagronomic analysis of the accessions and considering the economic criteria (mostly grain yield and earliness) used by farmers of northern Benin in the choice of sorghum landraces to grow in the current context of climate change [4, 24] twenty promising accessions were identified for utilization in sorghum improvement in Benin (Figure 4) and these also correspond to the 20 accessions gathered in group 2 earlier generated by the principal component analysis (Figure 2). The grain yield of these elite genotypes ranged from 4.85 to 10.57 tons/ha. The high grain yields were recorded in AL66 (10.57 tons/ha), AL57 (8.27 tons/ha), AT41 (7.85 tons/ha), and BO83 (7.66 tons/ha). The genotypes AT140, AT29, and AT14 recorded grain yield less than five tons per ha, respectively, 4.85, 4.85, and 4.89 tons/ha (Figure 4). The earliness (days to 50% flowering) varied between 57 and 189 days. Three genotypes,* namely*, AT29, AT41, and AT14 with respective yield 4.85, 7.85, and 4.89 tons/ha proved to be very early flowering at 57, 62, and 66 days, respectively. The highest days to flowering were recorded in AT17 and AT08. The genotype with the highest grain yield computed 138 days to flowering (Figure 4).

In terms of 100-seed weight five genotypes (AL64, AL66, AL63, AT19, and AT98) recorded >4 g seed weight (4.47, 4.22, 4.13, 4.04, and 4.02 grams, resp.). AT29, AT14, and AT41 were found to be short heighted or dwarf with 153.27, 168.67, and 180.37 cm height, respectively. Although AL66 had the highest grain yield and 100 seed weight (more than 4 grams) it was very tall (538.27 cm) and late maturing (138 days). In conclusion, AT41, AT14, and AT29 were found to possess genes for high grain yield, early maturing, and a medium plant height. Thus these were considered as super elite genotypes from existing sorghum germplasm from Benin and can be used towards national breeding programme.

## 4. Discussion

Agromorphological characterization is one of the most important steps towards effective utilization of existing diversity in a crop species towards its genetic improvement and classification of the germplasm [13, 25]. This study provides details of genetic variability and functional correlations among 142 sorghum germplasm collected from Northern Benin. The analysis was carried out using 10 qualitative and 14 quantitative agromorphological characters. Considerable variability was observed among the accessions for both types of phenotypic traits. The panicle compactness, glume colour and hairiness, and grain colour were the most variable qualitative traits. These characters were reported to be used extensively by the farmers in naming and identifying sorghum landraces [4]. On the other hand, number of grains per glume, grain shape dorsal view, grain covering, and threshability recorded less variation. It may be noted that unlike quantitative traits, qualitative characters are less influenced by the environment conditions. With this given fact such traits should not receive less importance in diversity studies as often this plays an important role in farmers' fields by influencing the varieties selection criteria.

The evaluated sorghum germplasm also showed wide variation for 14 quantitative characters analyzed. Observed variability for grain yield, 100-seed weight, and panicle weight were similar to earlier reports in sorghum [8, 12, 14] and maize [25, 26]. The results of this study showed that the majority of the Benin sorghum landraces had very tall height, large leaf area, and large number of internodes. Similar results were reported on Ethiopia sorghum landraces by Adugna [8]. Contrary to Ethiopian germplasm in which most landraces are relatively early maturing, Benin sorghum landraces studied are late maturing except three rare landraces which are AT14, AT41 and AT29 [24]. Nevertheless, the variability present in the grain yield can be used to derive grain sorghum and the high biomass (tall height, large leaf area, and large number of internodes) can be used as feed and fodder sorghum.

Application of PCA tool and multivariate statistical analysis provide useful means to estimate morphological diversity within and between germplasm collections [8, 20, 25, 26]. In this study, five axes contributed 73.8% of total diversity among the accessions. Earlier reports also suggested important contribution of first PCs in total variability while studying different traits [8, 20, 25, 26]. Accessions of G2 obtained using most discriminatory parameters can be involved in crossings to create maximum diversity among the lines [13]. This grouping can also help in defining minicore collection [13, 18] for sorghum in Benin.

The information regarding association among various characters is an important part for initiation of any breeding program as it provides an opportunity for the selection of genotypes having desirable traits simultaneously [16]. In the present experiment correlation analysis indicated some important associations among the quantitative traits studied. The days to 50% flowering showed positively correlation with LTL, PH, WTL, and NIN while it had negative association with ExL and PeL. Traits showing high and positive correlations with earliness should be emphasized for selecting early maturing genotypes. Besides, plant height showed positive association with stem third internode girth, panicle width, width of third leaf from top, length of third leaf from top, panicle length, and panicle weight which showed positive contribution to high grain yield that exhibited highly positive correlation with 100-seed weight. The positive correlation among these yield contributing traits suggested that these characters are important and are also good indicator for direct selection of high yielding genotypes. Similar results were reported by Elangovan et al. [12]; Abubakar and Bubuche [16]; and Kannababu et al. [27]. The width of third leaf from top and length of third leaf from top were highly associated as reported by Khaliq et al. [28]. These characters play a vital role in drought tolerance and will be useful for high biomass breeding in sorghum.

Both quantitative and qualitative traits were used to cluster the genotypes following UPGMA. However, it was observed that grouping based on quantitative traits alone clustered accessions with different morphotypes as identical (data not shown). Hence, focus was made on clustering based on qualitative traits alone (Figure 3). In the present study, 142 accessions of sorghum were classified into 89 morphotypes. This result indicated the existence of synonymies within the studied sorghum germplasm as reported by Dossou-Aminon et al. [4, 24]. However, current study suggested the need for breeders to evaluate germplasm with appropriate tools in order to characterize and evaluate accurately breeding germplasm materials into their distinct groupings and progression on their utility in the sorghum breeding program in the development of improved varieties. Nevertheless, the results of this study can be used for the development of new lines for other important abiotic and biotic traits such as drought and pests and diseases resistance.

From this agromorphological study, 20 promising genotypes of sorghum were identified based of their earliness and grain yield and correspond to 20 accessions obtained from ACP grouping. These criteria (earliness and grain yield) are mostly used by farmers in sorghum production zones to select cultivated landraces according to their economic importance [4, 24]. The identified genotypes that belong to different morphotypes (Figure 3) can be crossed in dialled mating design to increase the variability in breeding program [29]. Identified elite genotypes were subjected to three generations of mass selection to purify varieties for their quantitative useful traits such as yield and earliness according to Rakshit and Patil [18]. To enhance sorghum production in Benin these genotypes can be directly used due to their earliness and better suitability to the changing climate, mainly to unreliable rainfall in the region mostly affected by climate change [8, 24]. Therefore, they must be evaluated in different agroecologies to detect the superior genotypes adapted to each specific region and target environment. In addition, these early maturing varieties can be crossed to other high yielding genotypes like AL66.

## 5. Conclusion

This study highlighted the first information on the genetic diversity within the population of sorghum accessions of Northern Benin, which will be required for breeding program based on crosses of most promising accessions. The studied accessions showed high variability for both qualitative and quantitative traits. Pearson correlation coefficient analysis revealed significant positive association between economic traits that can be used in improvement of breeding activities in sorghum by breeders. The similarity cluster analysis facilitated the classification of the 142 accessions to 89 morphotypes. Three accessions were found to be most potential towards direct utilization in sorghum improvement. These may be further evaluated in multilocation trials. Some lines like AL66, AL57, BO83, AT41, AT29 and AL69 identified in the study can be involved in crossing programs to create maximum diversity for the benefit of new varieties' development. Molecular analysis of genetic diversity is recommended to optimize the use of the local sorghum genetic resources in varietal improvement.* In situ* and* ex situ* conservation programme should be implemented to reduce the genetic erosion.

## Figures and Tables

**Table 1:** Frequencies of the different phenotypes recorded within the various qualitative morphological parameters considered to describe the sorghum collection established.

Parameters and codes	Variables and score	Frequency (%)
Panicle compactness (PaCom)	Loose (1)	50
	Semi-loose (2)	19.72
	Semi-compact (3)	23.94
	Compact (4)	6.34

Peduncle shape (PeSh)	Erect (1)	76.76
	Semi-bent (2)	22.54
	Bent (3)	0.7

Glume colour (GlCol)	Beige (1)	2.82
	Brown (2)	4.93
	Purple (3)	38.03
	Black (4)	52.21
	Red (5)	2.02

Glume hairiness (GlHai)	Absent (1)	23.24
	Low (2)	59.86
	Medium (3)	9.15
	High (4)	7.75

Threshability (Thres)	Easy (1)	88.73
	Difficult (2)	11.27

Grain covering (GrCov)	50% (1)	90.14
	75% (2)	9.15
	100% (3)	0.7

Grain shape dorsal view (GrShDV)	Dimple (1)	92.96
	Convex (2)	7.04

Number of grain per glume (NuGrGl)	Single (1)	97.89
	Twin (2)	2.11

Grain shape profile view (GrShPV)	Circular (1)	22.54
	Elliptic (2)	77.46

Grain colour (GrCol)	White (1)	47.89
	Orange (2)	1.41
	Red (3)	47.89
	Yellow (4)	2.82

**Table 2:** Descriptive statistics of 14 quantitative agromorphological traits in 142 sorghum studied accessions.

Quantitative traits	Minimum	Maximum	Mean ± SE	Median	SD	CV
Days to 50% seed germination (d)	4	6	4.30 ± 0.04	4	0.52	12.06
Days to 50% flowering (d)	57	200	138.56 ± 2.92	137.50	34.79	25.11
Stem third internode girth (cm)	0.43	2.4	0.96 ± 0.02	0.91	0.28	28.56
Panicle width (cm)	3.83	18	9.13 ± 0.20	8.78	2.44	26.75
Exertion length (cm)	0	44.67	8.28 ± 0.73	5.40	8.73	105.52
Plant height (cm)	153.27	636.5	457.83 ± 7.87	470.67	93.76	20.48
Width of third leaf from top (cm)	4.4	10.27	7.52 ± 0.10	7.68	1.23	16.33
Length of third leaf from top (cm)	48.2	110.7	76.40 ± 0.88	76.32	10.53	13.78
Panicle length (cm)	17.13	62.23	38.88 ± 0.63	38.77	7.52	19.34
Peduncle length (cm)	28.47	93.33	51.49 ± 1.06	51.67	12.67	24.61
Panicle weight (g)	15	215.95	86.32 ± 2.78	83.03	33.10	38.35
100-seed weight (g)	2.01	4.47	3.25 ± 0.04	3.30	0.49	15.13
Number of internodes	7	38	22.49 ± 0.57	20.67	6.77	30.10
Grain yield (tons/ha)	0.72	10.57	3.15 ± 0.13	48.32	1.60	50.73

d = day, g = gram, SE = standard error of mean, SD = standard deviation, and CV **=** coefficient of variation.

**Table 3:** Principal component analysis (PCA) of different quantitative agromorphological traits in sorghum.

Variable	PC1	PC2	PC3	PC4	PC5
Days to 50% seed germination	0.100	0.071	0.016	−0.434	0.537
Days to 50% flowering	−0.285	−0.305	0.224	−0.309	−0.129
Stem third internode girth	−0.278	0.109	−0.301	0.415	0.169
Panicle width	−0.100	−0.080	−0.512	0.024	0.019
Exertion length	0.374	0.122	−0.158	−0.169	−0.186
Plant height	−0.317	−0.276	−0.133	−0.311	−0.220
Width of third leaf from top	−0.320	−0.076	−0.279	0.199	0.251
Length of third leaf from top	−0.111	−0.062	−0.117	0.064	0.259
Panicle length	−0.079	−0.042	−0.561	−0.302	−0.105
Peduncle length	0.334	0.093	−0.346	−0.288	−0.189
Panicle weight	−0.287	0.422	0.043	−0.135	−0.207
100-seed weight	−0.081	0.143	0.062	−0.363	0.563
Number of internodes	−0.369	−0.291	0.140	−0.189	−0.137
Grain yield	−0.248	0.495	0.036	−0.086	−0.118
Eigenvalue	**3.9343**	**2.8987**	**1.8203**	**1.3513**	**1.0655**
% of total variance	**26.2**	**19.3**	**12.1**	**9**	**7.1**
Cumulative variance (%)	**26.2**	**45.6**	**57.7**	**66.7**	**73.8**

**Table 4:** The correlation coefficients between agromorphological traits evaluated in Benin sorghum germplasm collection.

	50% SGD	50% FD	STING	PaW	ExL	PH	WTL	LTL	PaL	PeL	PaWt	100-SWt	NIN
50% FD	−0.035												
STING	−0.177^**^	−0.064											
PaW	−0.042	0.017	0.281^**^										
ExL	0.084	−0.439^**^	−0.341^**^	−0.065									
PH	−0.167^**^	0.606^**^	0.156^**^	0.184^**^	−0.369^**^								
WTL	−0.126^**^	0.202^**^	0.586^**^	0.288^**^	−0.411^**^	0.410^**^							
LTL	−0.071	0.120^*^	0.131^**^	0.055	−0.109^*^	0.150^**^	0.203^**^						
PaL	0.069	−0.011	0.180^**^	0.408^**^	−0.036	0.376^**^	0.199^**^	0.084					
PeL	0.154^**^	−0.413^**^	−0.299^**^	0.053	0.771^**^	−0.215^**^	−0.288^**^	−0.092	0.275^**^				
PaWt	−0.005	0.065	0.296^**^	−0.016	−0.210^**^	0.097^*^	0.172^**^	0.021	0.051	−0.193^**^			
100-SWt	0.176^**^	0.020	0.002	−0.039	−0.031	0.045	0.091	0.041	−0.006	−0.051	0.145^**^		
NIN	−0.156^**^	0.799^**^	0.147^**^	0.098^*^	−0.545^**^	0.750^**^	0.378^**^	0.101^*^	0.037	−0.519^**^	0.127^**^	0.039	
GY	−0.014	−0.086	0.317^**^	−0.034	−0.157^**^	−0.038	0.125^**^	0.014	0.022	−0.160^**^	0.901^**^	0.247^**^	−0.008

^**^ *P* ≤ 0.01; ^*^ *P* ≤ 0.05.

50% SGD = days to 50% seed germination (days), 50% FD = days to 50% flowering (days), STING = stem third internode girth (cm), PaW = panicle width (cm), ExL = exertion length (cm), PH = plant height (cm), WTL = width of third leaf from top (cm), LTL = length of third leaf from top (cm), PaL = panicle length (cm), PeL = peduncle length (cm), PaWt = panicle weight (g), 100-SWt = 100 seeds' weight (g), NIN = number of internodes, and GY = grain yield.
